# Clinical and Inflammatory Predictors of Neurocognitive Decline in Long COVID: A Two-Year Longitudinal Study with Propensity Score Matching

**DOI:** 10.3390/medicina62061180

**Published:** 2026-06-18

**Authors:** Iulia Elena Diaconu, Maria Ioana Onofrei, Andrei Vâță, Florin Manuel Roșu, Emilian Bogdan Ignat, Iulian Dan Cuciureanu, Mihnea Eudoxiu Hurmuzache, Mihaela Cătălina Luca

**Affiliations:** 1 Doctoral School, Grigore T. Popa University of Medicine and Pharmacy, 700115 Iasi, Romania; 2 Department of Infectious Diseases, Grigore T. Popa University of Medicine and Pharmacy, 700454 Iasi, Romania; maria-ioana.hunea@umfiasi.ro (M.I.O.);; 3 Clinic of Infectious Diseases, “Sf. Parascheva” Clinical Hospital of Infectious Diseases, 700116 Iasi, Romania; 4 Department of Neurology, Grigore T. Popa University of Medicine and Pharmacy, 700454 Iasi, Romania; 5 Department of Neurology, Clinical Rehabilitation Hospital, 700661 Iasi, Romania; 6 Department of Neurology, “Prof. Dr. N. Oblu” Clinical Emergency Hospital, 700309 Iasi, Romania

**Keywords:** vitamin D, Montreal cognitive assessment score, COVID-19, global atrophy scale, neutrophil-to-lymphocyte ratio, propensity score matching

## Abstract

*Background and Objectives*: Neurological complications of SARS-CoV-2 infection frequently impair patients’ long-term quality of life. This study aimed to identify clinical and laboratory risk factors—including inflammatory markers and micronutrients—for the occurrence or worsening of neurocognitive disorders in long COVID patients. *Materials and Methods*: In this prospective observational study, patients presenting with long COVID neurological manifestations were stratified by baseline MoCA score into two groups (≥23 and <23). Clinical, laboratory (inflammatory markers, 25-hydroxy vitamin D, vitamin B12, folic acid), and neuroimaging assessments (global cortical atrophy scale, Fazekas score) were performed over 24 months. Propensity score matching (PSM) for age, gender, and neurological comorbidities yielded 54 patients per group. *Results*: In the MoCA ≥ 23 group, significant predictors of cognitive decline included severe COVID-19 (OR = 2.211, 95% CI = 1.819–5.973, *p* = 0.012), autoimmune comorbidities (OR = 1.676, 95% CI = 1.191–2.390, *p* = 0.043), and elevated neutrophil-to-lymphocyte ratio (NLR; OR = 1.586, 95% CI = 1.431–2.122, *p* = 0.011). In the MoCA < 23 group, independent predictors were diabetes mellitus (OR = 3.021, 95% CI = 2.65–14.004, *p* = 0.016), autoimmune comorbidities (OR = 4.987, 95% CI = 1.412–6.033, *p* = 0.021), and NLR (OR = 5.944, 95% CI = 2.353–19.321, *p* = 0.015). Serum vitamin D levels were significantly associated with MoCA scores in both groups. *Conclusions*: COVID-19 severity, autoimmune comorbidities, NLR, and serum vitamin D represent key risk factors for neurocognitive decline in long COVID, highlighting potential targets for early intervention.

## 1. Introduction

The pandemic caused by the novel coronavirus SARS-CoV-2 has represented a major global public health challenge. Although mortality associated with COVID-19 was substantial, evidence accumulated rapidly and demonstrated that SARS-CoV-2 is not solely a respiratory pathogen but can also exert multisystemic effects, including neurological involvement [1].

Beyond the acute COVID-19, a considerable proportion of patients infected with SARS-CoV-2 develop a broad spectrum of long-term manifestations, including respiratory (e.g., lung fibrosis), cardiovascular (myocarditis, cardiac arrhythmia, thrombosis), metabolic (new-onset diabetes mellitus), neurological, and dermatological complications [2]. These persistent or newly occurring symptoms are encompassed within the definition of post-COVID syndrome, also referred to as long COVID syndrome [3,4]. A recent meta-analysis of 429 studies including more than 2 million patients reported a global pooled prevalence of manifestations of approximately 36% [5].

Long COVID neurological sequelae are highly heterogeneous and include headache, fatigue, “brain fog”, sleep disturbances, and long-term cognitive dysfunction (such as mood and behavioral changes, memory impairment, mental fatigue, and altered processing speed or executive function), as well as anxiety, depression, and stroke [6,7]. These manifestations occur independently of acute illness severity in a substantial proportion of patients [8].

Risk factors for neurological complications in the context of long COVID vary across studies. Several investigations and meta-analyses have indicated that female sex, pre-existing comorbidities, lack of COVID-19 vaccination, increased psychological stress, and abnormal body mass index (either obesity or low BMI) are more frequently associated with neurological manifestations and have been proposed as potential risk factors for symptoms such as headache and fatigue. Other studies have shown that severe acute infections requiring admission to the intensive care unit and mechanical ventilation further increase both the risk and the severity of cognitive impairment [5]. Additional determinants for these neurological manifestations include genetic susceptibility, environmental exposures, and socio-demographic factors, while the lockdown period and prolonged social isolation have been implicated in accelerating cognitive decline, particularly among patients with pre-existing dementia [8,9].

In a previous study conducted at our clinic, we observed that chronic fatigue syndrome was the most frequent neurological manifestation associated with long COVID-19 and was influenced primarily by patient age and, to a lesser extent, by serum vitamin D and vitamin B12 levels [10].

Therefore, the present study aimed to investigate the association between serum vitamin D levels and long COVID-related neurocognitive impairment, while also exploring additional clinical, inflammatory, and neuroimaging predictors of cognitive decline.

## 2. Materials and Methods

### 2.1. Study Design

This was a prospective observational longitudinal study conducted among patients with long–term neurological manifestations related to COVID-19, who were hospitalized in the Clinical Hospital of Infectious Diseases “Sf. Parascheva” Iasi, Romania. between January 2022 and December 2022 and were followed up for up to 2 years after SARS-CoV-2 infection.

**Figure 1:**
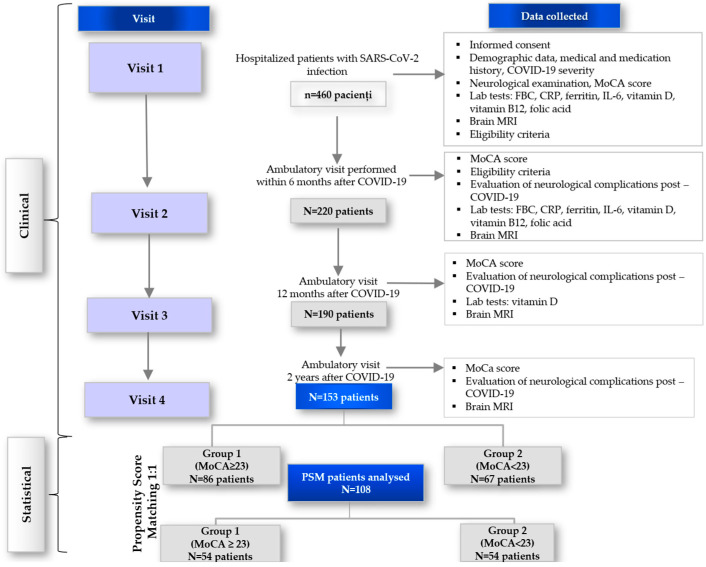
Flowchart of patient selection and statistics.

The study plan is described in Figure 1.

### 2.2. Study Population

Eligible patients were stratified in our study into two groups based on MoCA scores: ≥23 (normal cognitive function or mild cognitive impairment) and <23 (moderate–severe cognitive impairment).

#### 2.2.1. Inclusion Criteria

We included patients with confirmed SARS-CoV-2 infection (RT-PCR or validated antigen test), aged over 18 years, who survived to hospital discharge, and were available for follow-up at 6, 12, and 24 months after COVID-19. Only patients reporting persistent cognitive symptoms (e.g., brain fog, memory impairment, attention deficits) at the 6-month post-COVID evaluation were retained for longitudinal follow-up. Patients were required to undergo brain MRI, a neurological evaluation using the MoCA scale, and serum vitamin D measurements during SARS-CoV-2 infection, and to have provided written informed consent to participate in the study.

#### 2.2.2. Exclusion Criteria

Patients were excluded if they met any of the following conditions: pre-existing dementia or Alzheimer’s disease (based on MMSE, trial making test, brain CT, etc.) intellectual disability, or another chronic neurodegenerative disorder (e.g., Parkinson’s disease, atypical parkinsonism, Huntington’s disease); a central nervous system disease likely to affect cognition or neurological status (such as stroke with residual deficit, brain tumor, or moderate–severe traumatic brain injury); a major psychiatric disorder that significantly impaired cognition or the ability to participate in the study; a history of alcohol or psychoactive substance dependence; or sensory or motor impairments that precluded valid administration of the MoCA. Patients with SARS-CoV-2 reinfection and any acute infection at the six-month follow-up visit, or any condition that interfered with adequate understanding or completion of the study questionnaires were also excluded.

### 2.3. Data Collection

Figure 1 shows the details of the study procedures.

#### 2.3.1. COVID-19 History

Information on covariates was obtained from medical records and included sociodemographic characteristics (age, sex) and medical history (cardiovascular disease, diabetes, obesity, renal disease, oncologic or immunologic comorbidities). A detailed history of SARS-CoV-2 infection was also collected, including the severity of the acute episode, which was classified according to the WHO COVID-19 clinical progression scale [11].

#### 2.3.2. Neuropsychological Assessment

Neurological manifestations during COVID-19 were documented during hospitalization (Figure 1). Neurological assessment was carried out by two independent neurologists. The neurological anamnesis focused on the description of new-onset neurological manifestations (type and severity) and their impact on quality of life at each visit.

The Montreal cognitive assessment (MoCA) was administered at each time point (Figure 1). The MoCA score obtained at discharge (V1) was used to stratify patients. The MoCA is a 30-point screening instrument that evaluates naming, attention, concentration, orientation, language, short-term and working memory, verbal abstraction, visuospatial abilities, and executive function, and is widely used to detect cognitive impairment. Scores between 26 and 30 are generally considered normal; however, several cut-off values have been proposed in clinical studies, including 25 for mild cognitive impairment, 23 for cognitive impairment, and 21 for mild dementia [12,13].

Other neuropsychological tests were additionally performed to evaluate the patients’ cognitive status (trial making test B, verbal fluency, digit span, Addenbrooke’s cognitive examination-III, hospital anxiety and depression scales A and D). Addenbrooke’s cognitive examination-III (ACE_III test) is a comprehensive examination on attention, memory, verbal fluency, language and visuospatial skills.

#### 2.3.3. Laboratory Measurements and Systemic Inflammatory Markers

Serum 25-hydroxyvitamin D (ng/mL) levels were measured at visit 1, visit 2, and visit 3, whereas vitamin B12 (pg/mL) and folic acid were assessed at visit 1 and visit 2. All measurements were performed using a chemiluminescence immunoassay. The reference range for 25-hydroxyvitamin D was 6.23–49.9 ng/mL in females and 4.92–42.7 ng/mL in males, with the following categories: normal (>30 ng/mL), mild insufficiency (21–30 ng/mL), moderate insufficiency (10–20 ng/mL), and severe insufficiency (<10 ng/mL). The reference range for vitamin B12 was 197–771 pg/mL and for folic acid was 4.6–34.8 ng/mL.

Inflammatory markers included in the analysis, measured at visit 1 and visit 2, were C-reactive protein (CRP; mg/L), interleukin-6 (IL-6; pg/mL), ferritin (µg/L), and full blood count. Based on the full blood count, the following systemic inflammatory indices were calculated: neutrophil-to-lymphocyte ratio (NLR) and lymphocyte-to-monocyte ratio (LMR).

Full blood counts were performed using XS-1000i hematology analyzers (Sysmex, Hamburg, Germany). Biochemical parameters were measured on an Rx Imola analyzer (Randox, Antrim, UK). Vitamins and IL-6 were quantified using a CM-180 chemiluminescence immunoassay analyzer (Dirui, Changchun, China).

#### 2.3.4. Neuroimaging

All neuroimaging examinations were performed using up-to-date computed tomography and magnetic resonance imaging systems at each visit (Figure 1), in accordance with current clinical standards. MRI examinations were conducted on a Siemens MAGNETOM Sempra 1.5 T scanner (Siemens Healthineers, Forchheim, Germany), equipped with a 1.5 Tesla superconducting magnet and a 60 cm bore. The system utilizes Tim 4G RF technology, enabling high-resolution multiplanar imaging. All examinations followed standardized protocols to ensure consistency and diagnostic image quality.

For evaluation, the global cortical atrophy (GCA) scale and the Fazekas scale were used. The GCA is a four-point scale ranging from 0 (no atrophy) to 3 (severe atrophy). The Fazekas score ranges from 0 (normal) to 3 (severe) and grades the severity of white matter hyperintensities [14,15].

Image analysis was independently performed by two experienced neuroradiologists, who provided concordant ratings across all scales used in the study.

### 2.4. Endpoints

The primary endpoint was longitudinal neurocognitive decline, defined as a decrease of at least 2 points in the MoCA score at the final follow-up visit in patients with a history of SARS-CoV-2 infection [16]. The secondary outcomes were (i) to identify clinical and/or biological independent prognostic markers associated with long COVID-related neurocognitive impairment, as assessed by MoCA scores at pre-specified follow-up visits (V2, V3, and V4), and (ii) to evaluate the correlation between MoCA scores and MRI changes over time.

### 2.5. Statistical Analysis

All statistical analyses were performed using XLSTAT (Lumivero, Paris, France, version 2024) with a two-sided significance threshold set at *p* < 0.05.

#### 2.5.1. Descriptive Analysis

Continuous variables were assessed for distribution normality using the Shapiro–Wilk test and visual inspection of histograms and Q–Q plots. Continuous variables are reported as means, standard deviations, and coefficients of variation (%), whereas categorical variables are presented as frequencies (%). Between-group comparisons were performed using the Student’s *t*-test or Mann–Whitney U test for continuous variables, as appropriate, and the chi-square or Fisher’s exact test for categorical variables.

#### 2.5.2. Propensity Score Matching

To reduce confounding and improve comparability between patients stratified by baseline MoCA score (≥23 vs. <23), propensity score matching (PSM) was performed. The propensity score was estimated using a multivariate logistic regression model including the following covariates: age, sex, Charlson comorbidity index, and pre-existing neurological comorbidities. PSM was conducted using a logistic regression model with greedy nearest-neighbor matching based on Mahalanobis distance, incorporating the propensity score. Matching was carried out in a random 1:1 fashion, with a caliper width set at 0.1 × sigma.

#### 2.5.3. Regression Analysis

Multivariate logistic regression models were constructed to identify independent predictors (comorbidities, COVID-19 severity, NLR, LMR, vitamin D, vitamin B12, folic acid) of neurocognitive decline, defined as a decrease of ≥2 points in MoCA score at the final follow-up visit. Adjusted odds ratios (ORs) with 95% confidence intervals (CIs) were reported. Model discrimination was evaluated using the area under the receiver operating characteristic curve (AUC), and calibration was assessed using the Hosmer–Lemeshow goodness-of-fit test.

#### 2.5.4. Correlation Analysis

Associations between MoCA scores and neuroimaging parameters (GCA and Fazekas scores) were evaluated using Pearson or Spearman correlation coefficients, depending on data distribution.

## 3. Results

### 3.1. Characteristics of Participants

Analysis of our database comprising 460 hospitalized patients with SARS-CoV-2 infection revealed the following post-infection complications: cognitive impairment, including memory deficits and brain fog, in 47.83% of cases (*n* = 220); chronic fatigue in 45.21% (*n* = 208); headache in 38.70% (*n* = 178); dizziness in 36.3% (*n* = 167); depression in 11.09% (*n* = 51); tinnitus in 8.69% (*n* = 40); sleep disturbances in 6.08% (*n* = 28); extrapyramidal disorders in 5.21% (*n* = 24); stroke in 5.21% (*n* = 24); and paresthesia in 2.17% (*n* = 10).

Of the 220 eligible patients, 153 completed the study and were included in the final analysis. Among these, 56.21% (*n* = 86) had baseline MoCA scores ≥ 23 (group 1), while the remaining 43.79% (*n* = 67) scored below 23 (group 2).

In the unmatched cohort, we observed significant differences between groups in terms of age, cardiovascular and neurological comorbidities, Charlson index, severity of SARS-CoV-2 infection (grades 4 and 6) and occurrence of encephalopathy during the acute infection (Table 1). No gender differences were identified between the unmatched groups. PSM incorporated the following covariates: age, gender, Charlson index, other associated disorders (neurological), and COVID-19-associated encephalopathy.

The matched cohort comprised 108 patients (54 patients in each group), with a mean age of 63.67 ± 10.67 years in group 1 and 64.57 ± 9.19 years in group 2. After matching, no statistically significant differences remained for the matched covariates.

### 3.2. Analysis of Cognitive Status

The long-term association between COVID-19 and cognition (assessed by MoCA scores) was studied throughout the 2 years after SARS-CoV-2 hospitalization.

**Figure 2:**
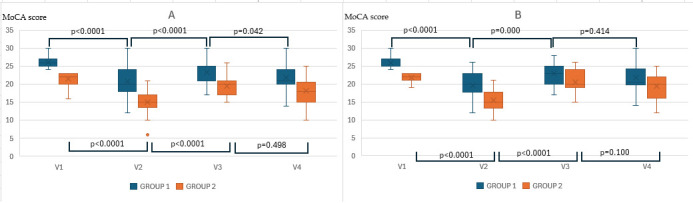
MoCA scores by group and visit—before PSM (**A**) and after PSM (**B**). V1—visit 1; V2—visit 2; V3—visit 3; V4—visit 4.

In the unmatched cohort, the incidence of cognitive decline in group 1 was 73.26% (*n* = 63), significantly lower than in group 2 (89.55%; *n* = 60) (*p* = 0.021). Significant differences between groups were noted at each visit. Both groups showed a significant (*p* < 0.05) decline in the MoCA score over the first 6 months after COVID-19, followed by an average increase of 3–4 points within the next 6 months. Overall, MoCA scores were significantly lower at each follow–up visit compared with baseline (Figure 2A).

After PSM, the incidence of cognitive decline in group 1 was 42.59% (*n* = 23), significantly lower than in group 2 (72.22%; *n* = 39) (*p* = 0.004). Significant differences between groups were noted at each visit. Both groups showed a significant (*p* < 0.05) decline in the MoCA score over the first 6 months after COVID-19, followed by an average increase of 2–4 points over the next 6 months. Overall, MoCA scores were significantly lower at each follow-up visit compared with baseline (Figure 2B).

**Figure 3:**
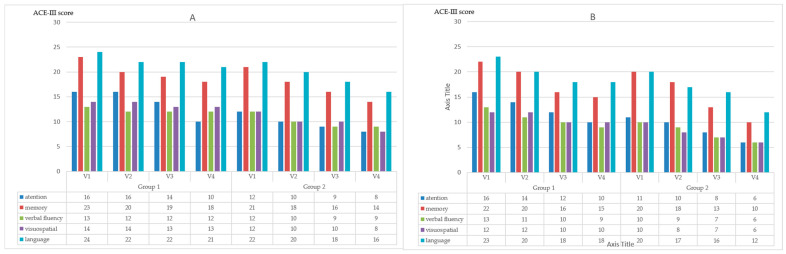
ACE-III mean scores by group and visit—before PSM (**A**) and after PSM (**B**). V1—visit 1; V2—visit 2; V3—visit 3; V4—visit 4.

The cognitive decline was confirmed by ACE-III results. Both before and after PSM, all domains of the ACE score were affected. For the memory task, the decrease was significant in 68.28% of cases from group 2 after PSM (Figure 3).

### 3.3. Analysis of Covariates

The prognostic potential of several factors (medical history of cardiovascular, neurologic, and autoimmune disorders; diabetes and obesity; NLR, LMR, IL-6, CRP, and ferritin) was evaluated.

Before PSM, the incidence of cardiovascular (*p* < 0.0001) and neurological (*p* = 0.004) disorders was higher in group 2 than in patients with MoCA score ≥ 23. Charlson score was significantly higher in group 2 (*p* < 0.0001).

Among laboratory tests, no significant differences between groups were found, except NLR (visit 2) and vitamin D (visit 1, visit 2) (Table 2).

Multivariate logistic regression analyses were performed to identify risk factors for a decline in MoCA scores 2 years after COVID-19.

Before PSM, in patients with MoCA score ≥ 23, the history of severe COVID-19 (OR = 2.473, 95% CI = 1.989–6.185, *p* = 0.001); neurological comorbidities (OR = 1.237, 95% CI = 1.102–5.136, *p* = 0.001); autoimmune comorbidities (OR = 1.103, 95% CI = 1.388–3.133, *p* = 0.046); ferritin measured 1 year after COVID-19 (OR = 0.998, 95% CI = 0.996–0.999, *p* = 0.010); NLR (OR = 1.770, 95% CI = 2.269–11.653, *p* = 0.005) and LMR (OR = 1.693, 95% CI = 1.126–1.829, *p* = 0.033); and vitamin D during (OR = 0.896, 95% CI = 0.834–0.962, *p* = 0.002) and within 6 months of COVID-19 (OR = 0.827, 95% CI = 0.747–0.916, *p* = 0.000) were significantly associated with lower MoCA scores (Table 3).

Before PSM, in patients with MoCA score < 23, diabetes mellitus (OR = 2.73, 95% CI = 1.914–8.114, *p* = 0.032); NLR (OR = 3.389, 95% CI = 2.448–5.624, *p* = 0.024) and LMR (OR = 1.200, 95% CI = 1.571–1.680, *p* = 0.029); and vitamin D during (OR = 0.889, 95% CI = 0.819–0.964, *p* = 0.005) and within 6 months of COVID-19 (OR = 0.878, 95% CI = 0.791–0.973, *p* = 0.013) were significantly associated with lower MoCA scores (Table 3).

After PSM, in patients with MoCA score ≥ 23, severe COVID-19 (OR = 2.211, 95% CI = 1.819–5.973, *p* = 0.012); autoimmune comorbidities (OR = 1.676, 95% CI = 1.191–2.390, *p* = 0.043); NLR (OR = 1.586, 95% CI = 1.431–2.122, *p* = 0.011) and LMR (OR = 1.074, 95% CI = 1.584–1.976, *p* = 0.018); and vitamin D during (OR = 1.250, 95% CI = 1.119–1.395, *p* < 0.0001) and measured within 6 months of COVID-19 (OR = 1.334, 95% CI = 1.157–1.537, *p* < 0.0001) were significantly associated with lower MoCA scores (Table 3).

After PSM, in patients with MoCA score < 23, diabetes mellitus (OR = 3.021, 95% CI = 2.65–14.004, *p* = 0.016); autoimmune comorbidities (OR = 4.987, 95% CI = 1.412–6.033, *p* = 0.021); NLR (OR = 5.944, 95% CI = 2.353–19.321, *p* = 0.015); and vitamin D during (OR = 1.327, 95% CI = 1.091–1.614, *p* = 0.005) and within 6 months of COVID-19 (OR = 1.415, 95% CI = 1.113–1.799, *p* = 0.005) were significantly associated with lower MoCA scores (Table 3).

### 3.4. Vitamin D, Vitamin B12, and Folic Acid Levels

In both cohorts, before and after propensity score matching (PSM), no statistically significant intergroup differences in vitamin D levels were identified at any visit; however, significant differences in the distribution of deficiency severity were observed. Prior to PSM, severe vitamin D deficiency was recorded in 2.99% of cases in group 2 (Table 2, Figure 2).

**Figure 4:**
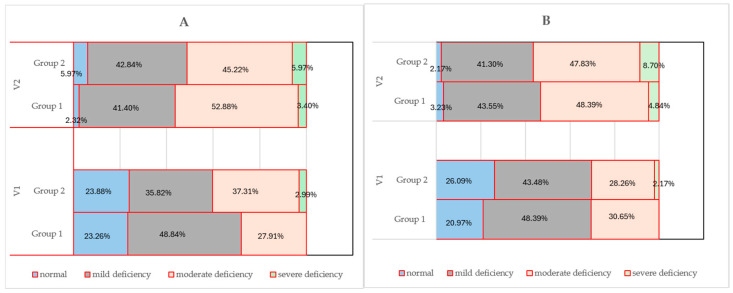
Percentage of patients with normal vitamin D levels and with vitamin D deficiency (mild, moderate, severe) by group and visit—before PSM (**A**) and after PSM (**B**). V1—visit 1; V2—visit 2.

Within 6 months after COVID-19 (visit 2), vitamin D levels were significantly lower in both the unmatched and matched cohorts (*p* < 0.001) when compared with visit 1. Prior to PSM, severe vitamin D deficiency (below 10 ng/mL) was recorded in 3.49% of cases with a MoCA score above 32, compared with 5.97% of cases with a MoCA score below 32. In the matched cohort, severe vitamin D deficiency was identified in 4.84% cases in group 1 versus 8.70% patients with a MoCA score below 23 (group 2) (Figure 4).

All patients received, between visit 2 and visit 3, vitamin D supplementation at a dose of 2000 IU daily for 1 year, after which significant increases and normalization of vitamin D levels (above 30 ng/mL) were observed in 100% cases at visit 2.

Irrespective of propensity score matching (PSM), no statistically significant within-group or intergroup differences in serum vitamin B12 or folic acid levels were observed after the acute SARS-CoV-2 infection compared with baseline values (Table 2).

### 3.5. Brain MRI Results

**Figure 5:**
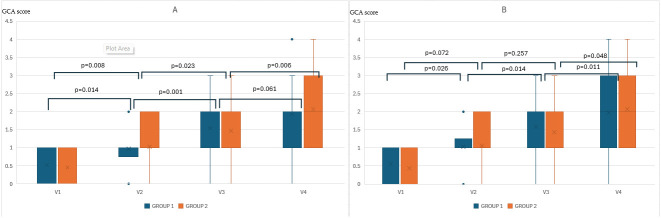
GCA scores by group and visit—before PSM (**A**) and after PSM (**B**). V1—visit 1; V2—visit 2; V3—visit 3; V4—visit 4.

In both the unmatched (Figure 5A) and matched (Figure 5B) cohorts, GCA and Fazekas scores increased progressively over the two-year follow-up period, with no statistically significant intergroup differences at any visit.

Before PSM, the highest progression of GCA and Fazekas scores was noted 1 year after COVID-19 (V3) in both groups: 47.67% cases (*n* = 41) for both scores in group 1 and 43.28% (*n* = 29) for GCA and 49.25% (*n* = 33) for Fazekas scores in group 2.

After PSM, the highest progression of GCA and Fazekas scores was noted 1 year after COVID-19 (V3) in both groups: 62.5% cases (*n* = 34) for GCA scores in group 1 and 70.83% (*n* = 39) in group 2.

These alterations were moderate but significantly correlated with MoCA score progression only in patients with MoCA < 23: Pearson coefficient = 0.34 (*p* = 0.01) for GCA score and Pearson coefficient = 0.27 (*p* = 0.04) for Fazekas score (Table 4).

## 4. Discussion

The present study identified newly developed or aggravated neurocognitive disorders following COVID-19 in a cohort of 153 patients followed for up to two years. After PSM (*n* = 108; 54 per group), the incidence of cognitive decline was 42.59% in group 1 (MoCA ≥ 23) and 72.22% in group 2 (MoCA < 23) (*p* = 0.004). Long COVID neurocognitive disorders are increasingly recognized as a significant manifestation after SARS-CoV-2 infection, with accumulating evidence demonstrating persistent deficits in attention, executive function, processing speed, and episodic memory, even among patients who did not experience critical illness.

**Figure 6:**
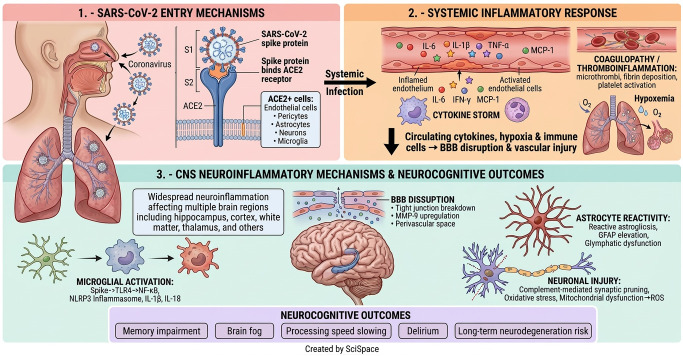
Neuroinflammation mechanisms in COVID-19 leading to neurocognitive impairment, adapted after [20,21,22,23,24,25,26].

The pathophysiology of long COVID cognitive impairment is considered multifactorial, comprising persistent systemic and neuroinflammation, endothelial and microvascular injury, blood–brain barrier disruption, and secondary neuronal and glial damage (Figure 6). SARS-CoV-2 infection triggers a systemic cytokine storm (with increased production of IL-6, IL-1β, TNF-α) that disrupts blood–brain barrier integrity through endothelial activation and metalloproteinase (MMP-9)-mediated tight junction breakdown, allowing inflammatory mediators to enter the central nervous system and initiate sustained neuroinflammation [17,18,19]. Within the central nervous system, microglial activation via spike protein–TLR4–NF-κB signaling and NLRP3 inflammasome engagement, reactive astrogliosis, complement-mediated synaptic pruning, and mitochondrial oxidative stress converge to produce hippocampal neuronal degeneration, reduced neurogenesis, and white matter microvascular injury [20,21,22,23]. These pathological processes manifest clinically as a spectrum of neurocognitive deficits—including episodic memory impairment, attention and executive dysfunction (brain fog), and psychomotor slowing—with emerging evidence suggesting a risk of accelerated long-term neurodegeneration in susceptible individuals (Figure 6) [24,25,26].

Neuroimaging and biomarker investigations have further demonstrated reductions in grey matter volume, most notably within the anterior cingulate cortex, alongside elevated markers of cerebral injury that correlate significantly with cognitive performance [27,28,29].

Longitudinal follow-up demonstrated persistent and, in some cases, progressive neurocognitive impairment following SARS-CoV-2 infection, as reflected by both MoCA trajectories and structural neuroimaging change (GCA and Fazekas scores).

In the present study, MoCA scores declined significantly in both groups, with the most pronounced deterioration at six months post-infection (group 1: 25.69 ± 1.51 → 21.08 ± 4.07; group 2: 21.86 ± 1.51 → 15.75 ± 2.92). The incidence of cognitive decline after PSM was 42.59% in group 1 and 72.22% in group 2 (*p* = 0.004). Objective assessments indicate that 20–50% of individuals exhibit measurable cognitive impairment at 6–12 months post-infection [30]. Notably, one large-scale longitudinal study reported that cognitive deficits observed one year after hospitalization were comparable in magnitude to approximately two decades of age-related cognitive decline, with changes particularly pronounced in psychomotor speed and semantic fluency. These deficits frequently correspond to patients’ subjective complaints of “brain fog,” mental fatigue, and word-finding difficulties, underscoring the clinical relevance of self-reported symptoms [28].

Neurocognitive disorders encompass a broad and heterogeneous spectrum of conditions varying considerably in nature and severity and their etiological diagnosis cannot be established based on a screening test alone. In accordance with current diagnostic recommendations, the evaluation of suspected Alzheimer’s disease or other neurocognitive disorders should follow a staged approach, beginning with clinical history, assessment of functional decline, neurological examination, cognitive screening, and neuropsychological testing, followed by structural neuroimaging and laboratory investigations to exclude potentially reversible or alternative causes. A wide array of standardized scales and neuropsychological instruments are available for the assessment of cognitive function, each validated for specific cognitive domains, including the mini-mental state examination (MMSE), the Montreal cognitive assessment (MoCA), the trail-making test, and the Rey auditory verbal learning test (RAVLT) [30]. Biomarker-based confirmation of Alzheimer’s disease requires evidence of Alzheimer pathology, most commonly through cerebrospinal fluid biomarkers, amyloid/tau PET, or validated blood-based biomarkers where available [31]. To exclude patients with significant cognitive disorders, we followed the guideline’s wave 0 (staging) and wave 1 (clinical syndromes) sections, which together encompass assessment of daily functioning and cognitive screening tests, neuropsychological evaluation, neuroimaging, and blood tests [31]. Therefore, in the present study, MoCA was used as a screening and longitudinal outcome measure of cognitive performance, not as a standalone diagnostic tool for Alzheimer’s disease. Patients with previously diagnosed dementia, Alzheimer’s disease, or other neurodegenerative disorders were excluded in order to reduce confounding and to focus on newly developed or aggravated post-COVID cognitive impairment.

MoCA score was selected as the primary outcome measure given its established sensitivity (approximately 80–90%) and the evidence supporting a cut-off of 23 for optimal balance of sensitivity (73.5%) and specificity (91.3%) in the evaluation of neurocognitive disorders [32,33]. Accordingly, our study cohort was stratified into two groups based on a MoCA cut-off value of 23. However, there are many factors that affect the interpretation of this test, including educational, demographic, social, medical (cardiac failure, chronic obstructive pulmonary disease, diabetes, metabolic syndrome, etc.) or drug (anticholinergics, proton pump inhibitors, NSAIDs, statins, chemotherapeutic agents, etc.) factors [34,35,36]. In addition, the severity of COVID-19, the duration of hospitalization, the need for oxygen supplementation, as well as anti-COVID-19 medication, can modify cognitive status, and thus, implicitly, the MoCA score [37]. Accordingly, the MoCA score obtained at hospital discharge was considered the reference baseline cognitive assessment for longitudinal comparisons. Other factors that could alter the MoCA score (especially concomitant medications) were noted, without any changes in chronic treatments during the study period compared with baseline data.

GCA is associated with cognitive decline and is observed in various neurodegenerative conditions [38]. Brain imaging studies have revealed structural abnormalities in patients with long COVID, including global cortical atrophy (GCA). In a prospective study of patients with pre-existing dementia who contracted COVID-19, significant increases in global cortical atrophy were observed one-year post-infection. This was accompanied by worsening of cognitive function across multiple domains, including attention, memory, fluency, language, and visuospatial abilities [39]. In the present study, GCA and Fazekas scores increased progressively over two years, with the highest progression at visit 3 (one-year post-infection): 62.5% of group 1 and 70.83% of group 2 patients showed GCA progression after PSM. GCA and Fazekas scores correlated significantly with MoCA score only in patients with moderate–severe cognitive impairment (GCA V3: r = 0.34, *p* = 0.01; Fazekas V3: r = 0.27, *p* = 0.04), suggesting structural brain changes have a greater functional impact where neuronal reserve is already reduced.

Advanced age, severe acute SARS-CoV-2 infection (necessitating hospitalization or intensive care unit admission), encephalopathy during the acute SARS-CoV-2 infection, and concurrent psychiatric comorbidities have been identified as consistent risk factors; nevertheless, clinically significant cognitive sequelae have also been documented in younger, previously healthy individuals [40].

Our study population comprised patients across a wide age range (21–78 years). Given that cognitive decline is strongly associated with ageing and pre-existing neurological conditions, propensity score matching (PSM) was applied to balance the two MoCA-defined groups on gender, age and pre-existing neurological comorbidities—the variables most likely to confound between-group comparisons of clinical and laboratory risk factors, including vitamin D, given that both independently determine circulating 25(OH)D levels and post-COVID cognitive trajectories. In contrast to previous studies, the present analysis demonstrated that neither patient age nor sex was significantly associated with deterioration of cognitive function after PSM, suggesting that once confounders related to age and comorbidity burden are controlled for, the severity of the acute illness itself emerges as a more decisive determinant of cognitive outcome.

In the present study, severe SARS-CoV-2 infection was associated with a 2.2- to 2.4-fold increase in the risk of a decline in MoCA score. The role of severe COVID-19 as a significant predictor of neurocognitive impairment has been corroborated by several independent investigations. In 56 hospitalized patients, longer duration of oxygen therapy was significantly associated with persistent cognitive impairment at 12 months (OR = 0.926, 95% CI [0.871–0.985], *p* = 0.015) [40]. Andriuta et al. (2022) noted that acute oxygen requirement was significantly associated with a composite neuropsychological summary score integrating measures of executive function, language, and processing speed (R^2^ = 0.319, *p* = 0.031) [41].

Among the associated diseases, cardiovascular and renal diseases, diabetes, obesity, and Alzheimer’s disease have been identified as negative predictors of COVID-19 neurocognitive complications [42,43]. In our study, diabetes mellitus was a significant predictor only in group 2 after PSM (OR = 3.021, 95% CI = 2.65–14.004, *p* = 0.016). Autoimmune comorbidities were independently associated with reduced MoCA scores in both groups after PSM: OR = 1.676 (95% CI = 1.191–2.390, *p* = 0.043) in group 1 and OR = 4.987 (95% CI = 1.412–6.033, *p* = 0.021) in group 2. The markedly stronger association in group 2 suggests that autoimmune dysregulation exerts a disproportionate impact on patients with pre-existing cognitive vulnerability. A large-scale investigation further demonstrated that the risk of developing long COVID syndrome was markedly elevated among patients with pre-existing autoimmune conditions, being 4.05-fold higher in those with Sjögren’s syndrome and 3.18-fold higher in those with rheumatoid arthritis (OR = 3.18; 95% CI: 0.99–10.46) [44]. In fact, studies have revealed that COVID-19 increased the risk for 11 new-onset autoimmune diseases (including diabetes, vasculitis, or inflammatory bowel disease) [45]. In a comprehensive analysis encompassing over one million patients with diverse autoimmune diseases, the risk of developing cognitive disorders, including dementia and Alzheimer’s disease, ranged from 0.96 (95% CI: 0.83–1.12) in patients with Hashimoto’s thyroiditis to 1.97 (95% CI: 1.88–2.07) in those with multiple sclerosis [46]. Therefore, the results obtained in our study are consistent with data from the literature, considering neurocognitive disorder as an aggravation of pre-existing autoimmune pathology.

The neutrophil-to-lymphocyte ratio (NLR), a well-established marker of systemic inflammation, has been shown to be a significant independent predictor of clinical outcomes in acute SARS-CoV-2 infection [47,48]. Furthermore, NLR has been established as a clinically relevant diagnostic and prognostic biomarker across a spectrum of infectious and non-infectious neurological conditions, including cerebrovascular disease, intracerebral hemorrhage, craniocerebral trauma, and primary or metastatic brain tumors [47,48,49,50,51,52,53,54]. In the present study, NLR was identified as a significant independent predictor of cognitive decline in both groups after PSM: OR = 1.431 (95% CI = 1.586–2.122, *p* = 0.011) in group 1 and OR = 5.944 (95% CI = 2.353–19.321, *p* = 0.015) in group 2. The substantially higher OR in group 2 indicates that sustained systemic inflammation has a disproportionately greater prognostic impact in patients who are already present with cognitive impairment at baseline. The lymphocyte-to-monocyte ratio (LMR) also showed a significant association in group 1 after PSM (OR = 1.584, 95% CI = 1.074–1.976, *p* = 0.018), providing additional evidence that the balance between innate and adaptive immune responses is a key determinant of cognitive outcomes in long COVID. In contrast, IL-6, CRP, and ferritin were not independently significant after PSM, suggesting that NLR and LMR are more sensitive markers of neurologically relevant inflammation in this context.

We selected vitamin D, vitamin B12, and folic acid as covariates in our study. These vitamins are essential for normal neurological function, with pleiotropic effects on immune regulation, neurotransmitter synthesis, myelin maintenance, oxidative stress defense, and mitochondrial energy metabolism [55,56].

The neuroprotective mechanisms of vitamin D are well-characterized and include modulation of regulatory T cell differentiation, suppression of pro-inflammatory cytokine production, upregulation of antioxidant enzymes, and direct effects on neurotransmitter synthesis—particularly serotonin and dopamine pathways. Vitamin D receptors are expressed in brain regions critical for cognition and mood regulation, including the hippocampus, prefrontal cortex, and amygdala [57,58,59]. These mechanisms are directly relevant to the neuroinflammatory and oxidative stress pathways implicated in long-COVID cognitive impairment [60]. The effects of vitamin D are due to its active metabolite, 25-hydroxy-vitamin D, which is subsequently converted to 1,25-dihydroxyvitamin D (active metabolite) and 24,25-dihydroxyvitamin D (metabolite considered inactive). We did not use 1,25-dihydroxyvitamin D in our study, despite its clinical value, because it has a shorter half-life (4–6 h) and because of analytical difficulties and uncertainties in its determination methods [61,62]. In the present study, serum 25 hydroxy-vitamin D levels were significantly lower at six months post-infection (visit 2): group 1: 19.54 ± 6.04 ng/mL vs. 25.11 ± 7.46 ng/mL (baseline); group 2: 16.00 ± 4.86 ng/mL vs. 20.76 ± 6.42 ng/mL (baseline); *p* = 0.023. All patients received, between visit 2 and visit 3, vitamin D supplementation at 2000 IU/day for one year, resulting in normalization of levels (>30 ng/mL) in 100% of cases by visit 3. A high prevalence of vitamin D deficiency among long COVID populations has been consistently reported across multiple studies. In a cross-sectional investigation of 82 patients with long COVID presenting with fatigue or neuropsychiatric symptoms, Charoenporn et al. (2024) reported that 73.2% of cases met the criteria for vitamin D deficiency (defined as <20 ng/mL), 23.2% exhibited insufficiency (20–30 ng/mL), and only 3.6% demonstrated adequate vitamin D concentrations [63]. In a study of 125 adults with long COVID, Guerrero-Romero et al. (2023) [64] demonstrated that 25-hydroxyvitamin D deficiency (<30 ng/mL) was highly prevalent and independently associated with hypomagnesemia, with an adjusted odds ratio of 3.1 (95% CI: 2.3–12.4; *p* = 0.005). Patients presenting with concurrent vitamin D deficiency and hypomagnesemia exhibited a significantly greater burden of clinical manifestations, encompassing fatigue, memory impairment, attentional disorders, and depressive symptoms [64]. Among mild COVID-19 outpatients presenting with subacute neurological sequelae, Taskiran-Sag et al. (2022) [65] reported a mean serum vitamin D level of 20.9 ± 2.7 ng/mL, indicating widespread insufficiency. Notably, no statistically significant association was identified between vitamin D concentrations and either the presence or severity of neurological symptoms, suggesting that the underlying relationship may be more complex than a straightforward dose–response correlation [65].

Fernandes et al. (2022) [66] reported baseline serum 25-hydroxyvitamin D levels of 21.8 ± 10.7 ng/mL in hospitalized COVID-19 patients, with 14.1% fulfilling the criteria for severe deficiency (<10 ng/mL). Collectively, these findings indicate that vitamin D deficiency is prevalent during the acute phase of COVID-19 and may persist in the post-acute recovery period [66].

In our study, vitamin D emerged as a statistically significant predictor of cognitive decline in both groups, with a consistent and noteworthy directional shift between the unmatched and matched cohorts. Before PSM, vitamin D measured during acute COVID-19 and within six months thereafter was negatively associated with cognitive decline in both groups—group 1 (MoCA ≥ 23): OR = 0.896 (95% CI = 0.834–0.962, *p* = 0.002) and OR = 0.827 (95% CI = 0.747–0.916, *p* < 0.001); group 2 (MoCA < 23): OR = 0.889 (95% CI = 0.819–0.964, *p* = 0.005) and OR = 0.878 (95% CI = 0.791–0.973, *p* = 0.013)—suggesting a protective effect of higher vitamin D levels. Following PSM, however, this association reversed direction: vitamin D became positively associated with cognitive decline in both groups—group 1: OR = 1.250 (95% CI = 1.119–1.395, *p* < 0.0001) and OR = 1.334 (95% CI = 1.157–1.537, *p* < 0.0001); group 2: OR = 1.327 (95% CI = 1.091–1.614, *p* = 0.005) and OR = 1.415 (95% CI = 1.113–1.799, *p* = 0.005)—indicating that higher vitamin D levels at these timepoints were independently associated with greater risk of cognitive deterioration within matched groups. This apparent discrepancy between the pre- and post-PSM findings is best explained as a confounding artifact in the unmatched cohort. Before matching, the two groups were unbalanced with respect to gender and pre-existing neurological comorbidity burden—both variables that independently determine circulating vitamin D levels and post-COVID cognitive trajectories. Specifically, female sex is associated with higher rates of post-COVID neurological symptoms and cognitive impairment, while simultaneously exhibiting sex-hormone-mediated differences in vitamin D metabolism and binding protein regulation [67,68].

In the unmatched cohort, these imbalances generated a spurious protective association for vitamin D, whereby patients with higher vitamin D levels were also those with fewer neurological comorbidities and more favorable sex-related profiles—independently conferring better cognitive outcomes. PSM on gender and neurological comorbidities eliminated this confounding, unmasking the true within-group relationship. The post-PSM risk direction likely reflects reverse causation: patients with greater systemic inflammatory burden—evidenced by markedly elevated NLR in both groups—may exhibit transiently higher measured 25(OH)D as a consequence of inflammatory metabolic shifts, while the same inflammatory state drives cognitive deterioration. In this context, vitamin D measured during and shortly after acute COVID-19 may serve as a biomarker of inflammatory activity rather than an independent neuroprotective factor, a pattern consistent with the broader literature that questions the causal role of vitamin D in cognitive preservation [69].

More than five years after the onset of the COVID-19 pandemic, the long-term sequelae of SARS-CoV-2 infection continue to be investigated. Long COVID represents a complex multisystem disorder in which persistent systemic inflammation and immunosenescence are recognized as central pathophysiological mechanisms [70,71]. A growing body of research focuses on identifying prognostic factors that may inform therapeutic strategies to attenuate these pathophysiological processes and mitigate the risk of long-term complications of long COVID, including neurocognitive impairment.

**Limitations of the study**. The present study is subject to several limitations. First, the single-center design and relatively small sample size may limit the generalizability of the findings. Although PSM was employed to minimize confounding bias, this approach further reduced the available sample size. Additionally, several potentially relevant risk factors were not incorporated into the analysis, including the number of SARS-CoV-2 reinfections, the viral strain, and the patient’s vaccination status.

The unavailability of CSF markers, DaT-SCAN, and PET for the definitive diagnosis of other neurodegenerative pathologies, and the reliance on cognitive screening tests, may introduce confounding factors into our analysis. Although multiple cognitive assessments were utilized for patient screening, the Montreal cognitive assessment (MoCA) score served as the primary criterion for categorizing subjects into two groups. The interpretation of results based on the MoCA score may be influenced by potential confounding factors. Furthermore, vitamin B12 and folic acid levels were measured primarily for screening purposes. As the values obtained in our cohort remained within normal reference ranges, repeat testing was not conducted during the study period. Consequently, it was not possible to determine the potential longitudinal impact of changes in these parameters on long-term cognitive outcomes. The disproportionate distribution of males and females in the study cohort, and the wide range of age distribution between different groups before and after PSM, could also affect the interpretation of the results.

Larger multicenter studies with expanded patient cohorts and better-defined eligibility criteria will be performed to confirm and validate these findings.

## 5. Conclusions

The findings of the present study indicate that, depending on the MoCA score, several clinical and laboratory parameters—including history of severe COVID-19, autoimmune comorbidities, neutrophil-to-lymphocyte ratio, and serum 25-hydroxyvitamin D concentration—are significant prognostic indicators for cognitive impairment following COVID-19. These findings support routine monitoring of NLR and vitamin D status during post-COVID follow-up as part of a risk-stratification approach for neurocognitive complications.

## Figures and Tables

**Table 1:** Descriptive characteristics of patients.

Parameter	BEFORE PSM			AFTER PSM		
	Group 1 (*n* = 86)	Group 2 (*n* = 67)		Group 1 (*n* = 54)	Group 2 (*n* = 54)	
	Mean ± Dev Std (CV%)	Mean ± Dev Std (CV%)	*p*-Value	Mean ± Dev Std (CV%)	Mean ± Dev Std (CV%)	*p*-Value
Age (years)	49.54 ± 12.44 (25.0)	63.61 ± 9.08 (14.0)	<0.0001	63.67 ± 10.67 (16.75)	64.57 ± 9.19 (14.23)	0.828
Charlson index	2.04 ± 1.97 (97.0)	4.13 ± 1.85 (45.0)	<0.0001	4.33 ± 1.67 (38.57)	4.33 ± 1.52 (35.13)	0.911
MoCA score V1	26.07 ± 1.72 (7.0)	21.81 ± 1.16 (5.1)	<0.0001	25.69 ± 1.51(5.89)	21.86 ± 1.51 (6.89)	<0.0001
MoCA score V2	20.74 ± 4.10 (2.0)	15.41 ± 3.04 (19.72)	<0.0001	21.08 ± 4.07 (19.31)	15.75 ± 2.92 (18.56)	<0.0001
MoCA score V3	23.44 ± 2.75 (12.0)	19.25 ± 2.67 (13.87)	<0.0001	23.38 ± 2.78 (19.31)	19.39 ± 2.60 (13.39)	<0.0001
MoCA score V4	21.88 ± 3.30(15.10)	17.12 ± 2.05 (11.97)	<0.0001	21.21 ± 2.45 (11.55)	18.75 ± 3.46 (18.45)	0.01
	Percentage (%)	Percentage (%)	*p*-value	Percentage (%)	Percentage (%)	*p*-value
Gender						
Female	64.10	54.39	0.337	63.79	46.87	0.183
Male	35.90	45.61	0.337	36.21	53.12	0.183
Comorbidities						
Cardiovascular	38.46	91.23	<0.0001	45.83	62.5	0.385
Diabetes	30.77	43.86	0.167	45.83	50.0	0.561
Neurologic	31.58	46.30	0.004	22.86	21.87	0.148
Autoimmune	26.92	47.09	0.063	29.17	25.00	0.68
Obesity	53.84	47.36	0.569	50.0	62.5	0.56
COVID-19 history						
COVID-19 severity (WHO scale)						
4.00	39.74	17.54	0.010	16.67	19.23	0.967
5.00	52.56	59.65	0.520	20.83	22.50	0.973
6.00	25.39	22.81	0.025	18.33	20.00	0.464
Encephalopathy	16.67	42.11	0.002	29.17	20.12	0.687

MoCA, Montreal cognitive assessment; WHO, World Health Organization.

**Table 2:** Descriptive statistics of laboratory results.

	BEFORE PSM							AFTER PSM						
Parameter	Group 1 (*n* = 86)			Group 2 (*n* = 67)				Group 1 (*n* = 54)			Group 2 (*n* = 54)			
	Mean	St. Dev.	CV%	Mean	St. Dev.	CV%	*p*-Value	Mean	St. Dev.	CV%	Mean	St. Dev.	CV%	*p*-Value
NLR V1	6.63	5.47	96.67	9.00	8.12	90.22	0.096	8.98	6.39	71.07	8.30	7.05	84.93	0.397
NLR V2	1.44	2.32	82.53	8.02	7.69	95.86	0.825	2.53	3.64	143.90	4.28	3.30	77.13	0.001
LMR V1	2.70	1.95	72.36	2.45	2.38	96.94	0.133	3.01	2.25	74.84	2.74	2.31	84.06	0.566
LMR V2	2.66	2.34	87.98	2.59	2.43	93.63	0.373	2.54	1.66	65.38	2.29	1.73	75.55	0.709
IL-6 V1 (pg/mL)	1478.81	1091.76	61.78	1456.32	1205.55	82.78	0.634	1188.35	1101.55	92.70	1324.17	1063.74	80.33	0.629
IL-6 V2 (pg/mL)	110.64	68.36	47.41	89.94	64.59	71.82	0.733	115.23	71.13	61.72	106.19	70.19	66.10	0.626
Ferritin V1 (µg/L)	787.69	373.42	43.47	853.23	354.89	41.59	0.674	709.48	371.19	52.32	750.13	369.11	49.21	0.820
Ferritin V2 (µg/L)	689.34	299.68	51.15	638.85	296.34	46.39	0.069	640.95	309.60	48.30	668.40	321.44	48.09	0.847
CRP V1 (mg/L)	97.53	49.88	56.81	107.48	43.76	40.71	0.846	100.22	55.20	55.08	102.48	44.17	43.10	0.891
CRP V2 (mg/L)	77.23	43.87	56.72	83.70	44.91	53.65	0.543	67.61	42.06	62.21	79.44	45.77	57.62	0.358
Vitamin B12-V1 (pg/mL)	529.87	187.91	35.46	514.55	193.50	37.61	0.954	580.00	189.66	32.70	523.57	198.76	37.96	0.368
Vitamin B12-V2 (pg/mL)	573.15	220.66	38.50	641.29	209.96	32.74	0.136	646.80	191.23	29.57	607.25	210.34	34.64	0.520
Folic acid V1 (ng/mL)	33.08	28.55	42.43	23.59	19.78	40.97	0.664	9.34	3.67	39.27	9.73	3.36	34.55	0.666
Folic acid V2 (ng/mL)	31.32	46.18	37.41	20.03	35.86	34.51	0.533	10.03	4.58	45.63	10.81	4.37	40.44	0.403
Vitamin D V1 (ng/mL)	25.35	7.63	30.10	25.27	8.35	33.04	0.256	25.11	7.46	29.72	20.76	6.42	30.92	0.038
Vitamin D V2 (ng/mL)	19.50	5.87	30.10	19.44	6.42	33.04	0.256	19.54	6.04	30.92	16.00	4.86	30.38	0.023
Vitamin D V3 (ng/mL)	62.36	10.86	17.42	67.65	9.61	14.20	0.096	65.60	9.86	15.02	64.29	10.11	15.73	0.607

PSM, propensity score matching; NLR, neutrophil-to-lymphocyte ratio; LMR, lymphocyte-to-monocyte ratio; IL-6, interleukin-6; CRP, C-reactive protein.

**Table 3:** Multivariate logistic regression analysis.

	BEFORE PSM						AFTER PSM					
Source	Group 1 (*n* = 86)			Group 2 (*n* = 67)			Group 1 (*n* = 54)			Group 2 (*n* = 54)		
	OR	95% CI	*p*-Value	OR	95% CI	*p*-Value	OR	95% CI	*p*-Value	OR	95% CI	*p*-Value
Age	1.016	0.978–1.055	0.411	0.973	0.923–1.027	0.324	1.021	0.977–1.067	0.353	0.988	0.914–1.069	0.764
Gender	0.984	0.965–1.002	0.082	0.913	0.317–2.629	0.866	0.451	0.148–1.370	0.160	0.875	0.214–3.583	0.853
COVID-19 severity	2.473	1.989–6.185	0.035	0.955	0.376–2.430	0.924	2.211	1.819–5.973	0.012	0.861	0.310–2.397	0.775
COVID-19 encephalopathy	0.095	0.018–0.505	0.006	0.728	0.241–2.197	0.573	0.261	0.048–1.427	0.121	0.712	0.168–3.022	0.645
Cardiovascular comorbidities	1.025	0.372–2.821	0.962	0.436	0.062–3.068	0.405	1.259	0.404–3.923	0.691	0.433	0.035–5.411	0.516
Diabetes	0.878	0.310–2.486	0.806	2.723	1.914–8.117	0.032	0.970	0.285–3.295	0.961	3.021	2.652–4.004	0.016
Neurological comorbidities	1.237	1.102–5.136	0.001	0.786	0.254–2.428	0.676	1.410	1.203–9.801	0.728	0.635	0.116–3.478	0.600
Autoimmune comorbidities	1.388	1.103–3.133	0.046	1.224	1.088–5.282	0.017	1.676	1.191–2.390	0.043	4.987	1.412–6.033	0.021
Charlson index	0.975	0.776–1.227	0.832	1.040	0.790–1.370	0.778	1.122	0.861–1.461	0.395	1.079	0.740–1.575	0.693
Ferritin-V1	1.000	0.999–1.001	0.734	1.000	0.999–1.001	0.914	1.000	0.999–1.002	0.504	1.000	0.998–1.002	0.978
IL-6-V1	1.000	0.999–1.000	0.181	1.000	0.999–1.000	0.139	1.000	0.999–1.001	0.949	0.999	0.998–1.000	0.055
NLR-V1	2.269	1.770–11.653	0.005	3.389	2.448–5.624	0.024	1.586	1.431–2.122	0.011	5.944	2.353–9.321	0.015
LMR-V1	1.693	1.126–1.829	0.033	1.571	1.200–1.680	0.029	1.584	1.074–1.976	0.018	1.078	0.754–1.542	0.679
CRP-V1	1.015	1.001–1.029	0.035	0.995	0.984–1.007	0.446	1.003	0.993–1.013	0.534	1.009	0.992–1.026	0.307
Folic acid-V1	0.930	0.822–1.052	0.246	0.968	0.832–1.126	0.673	0.888	0.765–1.032	0.121	0.991	0.812–1.210	0.930
Vitamin B12-V1	1.002	0.999–1.004	0.177	0.997	0.994–1.000	0.068	1.001	0.998–1.004	0.447	0.997	0.993–1.002	0.211
Vitamin D-V1	0.896	0.834–0.962	0.002	0.889	0.819–0.964	0.005	1.250	1.119–1.395	<0.0001	1.327	1.091–1.614	0.005
Vitamin D-V2	0.827	0.747–0.916	0.000	0.878	0.791–0.973	0.013	1.334	1.157–1.537	<0.0001	1.415	1.113–1.799	0.005

PSM, propensity score matching; NLR, neutrophil-to-lymphocyte ratio; LMR, lymphocyte-to-monocyte ratio; IL-6, interleukin-6; CRP, C-reactive protein.

**Table 4:** Pearson correlation test between MoCA and GCA and Fazekas scores.

	Before PSM				After PSM			
	Group 1		Group 2		Group 1		Group 2	
	Pearson Coefficient	*p*-Value	Pearson Coefficient	*p*-Value	Pearson Coefficient	*p*-Value	Pearson Coefficient	
GCA V1	0.07	0.58	0.30	0.16	0.06	0.75	0.04	0.75
GCA V2	0.23	0.09	0.30	0.16	0.01	0.98	0.22	0.11
GCA V3	0.21	0.11	0.37	0.08	0.29	0.13	0.34	0.01
GCA V4	0.21	0.13	0.06	0.78	0.31	0.11	0.13	0.34
Fazekas V1	0.16	0.24	0.18	0.41	0.14	0.48	0.20	0.15
Fazekas V2	0.16	0.25	0.13	0.56	0.16	0.41	0.14	0.29
Fazekas V3	0.25	0.06	0.42	0.04	0.15	0.45	0.27	0.04
Fazekas V4	0.09	0.52	0.13	0.56	0.16	0.41	0.18	0.19

V1—visit 1; V2—visit 2; V3—visit 3; V4—visit 4; PSM, propensity score matching; GCA—global cortical atrophy scale.

## Data Availability

The data presented in this study are available on request from the corresponding author. The requests for accessing individual data will be evaluated by the research team, and the ethics committees, to ensure that the data will be used only for research purposes, as well as to ensure data protection.

## Abbreviations

The following abbreviations are used in this manuscript:

ACE-2	Angiotensin-Converting Enzyme type 2
CCL 2	C-C motif chemokine ligand 2
CI	Confidence interval
CRP	C-reactive protein
CNS	Central nervous system
CV	Coefficient of variation
CXCL10	C-X-C motif chemokine ligand 10
FBC	Full blood count
GCA	Global atrophy scale
IL-1β	Interleukin-1β
IL-6	Interleukin-6
IL-18	Interleukin-18
IFNγ	Interferon γ
LMR	Lymphocyte-to-monocyte ratio
MCP-1	Monocyte chemoattractant proteins -1
MoCA	Montreal cognitive assessment
NLR	Neutrophil-to-lymphocyte ratio
OR	Odds ratio
PIV	Pan-immune-inflammation index
PSM	Propensity score matching
ROS	Reactive oxygen species
SOD	Superoxide dismutase
TNF-α	Tumor necrosis factor -α
TLR4	Toll-like receptor 4
V1/V2/V3/V4	Visit 1/visit 2/visit 3/visit 4
WHO	World Health Organization
